# Efficient residual network using hyperspectral images for corn variety identification

**DOI:** 10.3389/fpls.2024.1376915

**Published:** 2024-04-16

**Authors:** Xueyong Li, Mingjia Zhai, Liyuan Zheng, Ling Zhou, Xiwang Xie, Wenyi Zhao, Weidong Zhang

**Affiliations:** ^1^ School of Computer Science and Technology, Henan Institute of Science and Technology, Xinxiang, China; ^2^ School of Information Engineering, Henan Institute of Science and Technology, Xinxiang, China; ^3^ School of Information Science and Technology, Dalian Maritime University, Dalian, China; ^4^ School of Artificial Intelligence, Beijing University of Posts and Telecommunications, Beijing, China

**Keywords:** crop variety, hyperspectral image, channel attention, linear discriminant analysis, deep learning

## Abstract

Corn seeds are an essential element in agricultural production, and accurate identification of their varieties and quality is crucial for planting management, variety improvement, and agricultural product quality control. However, more than traditional manual classification methods are needed to meet the needs of intelligent agriculture. With the rapid development of deep learning methods in the computer field, we propose an efficient residual network named ERNet to identify hyperspectral corn seeds. First, we use linear discriminant analysis to perform dimensionality reduction processing on hyperspectral corn seed images so that the images can be smoothly input into the network. Second, we use effective residual blocks to extract fine-grained features from images. Lastly, we detect and categorize the hyperspectral corn seed images using the classifier softmax. ERNet performs exceptionally well compared to other deep learning techniques and conventional methods. With 98.36% accuracy rate, the result is a valuable reference for classification studies, including hyperspectral corn seed pictures.

## 1 Introduction

The cultivation of maize holds significant economic importance as a pivotal crop. As automation technology becomes increasingly prevalent in the agricultural sector, a growing need for automated classification and identification of corn seeds is needed. Accurately identifying corn seeds is vital for effective planting management, variety enhancement, and quality control of agricultural products (ElMasry et al., 2019). However, traditional manual classification methods can be inefficient and require substantial human resources. In the agricultural field, hyperspectral imaging technology has found extensive application (Zhang et al., 2022). Hyperspectral images offer multi-band spectral data and capture more comprehensive plant information than traditional RGB images (Wang et al., 2019; Ahmad et al., 2021). Therefore, hyperspectral imaging technology is widely employed in non-destructive testing of crop seed varieties, quality assessment, and vigor analysis (Ma et al., 2020; Zhang et al., 2023a; Zhang et al., 2024a). Nevertheless, the high-dimensional nature of hyperspectral data, complex features, noise, and variations in illumination poses challenges for traditional image processing and classification techniques in recognizing hyperspectral corn seed images (Zhang et al., 2021a; Ghaderizadeh et al., 2022; Huang et al., 2022). Hence, this article aims to enhance corn seed hyperspectral image recognition accuracy and efficiency using the efficient residual network (ERNet).

ERNet is an image classification and recognition model based on deep learning. First, preprocessing and feature extraction were performed on hyperspectral image data of different varieties and qualities of corn seeds. Next, the ERNet model is constructed, trained, and optimized to learn the image’s feature representation and classification decision. Finally, the performance and effectiveness of the proposed method will be evaluated, compared, and analyzed with traditional image classification methods.

ERNet enhances model performance and efficiency by incorporating residual connections and lightweight attention mechanism. It leverages collaborative learning strategies among different modules to effectively exploit coarse-grained, fine-grained, and abstract-level features. By fully utilizing the feature extraction capabilities of deep networks, ERNet overcomes the challenges of gradient disappearance and information loss in deep networks, enabling improved learning and image feature extraction. Compared to traditional deep convolutional neural networks, ERNet offers advantages such as reduced parameter count, high computational efficiency, and suitability for processing high-dimensional image data. The critical contributions of the proposed ERNet model in this study can be summarized as:

- We propose ERNet, an efficient residual network specifically designed for identifying corn varieties using hyperspectral data. ERNet leverages the power of residual connections and lightweight attention mechanism to address issues like gradient disappearance and reducing information loss commonly encountered in deep networks. As a result, it dramatically enhances the model’s performance and efficiency, leading to more accurate and efficient corn variety identification.
- We introduce two efficient residual modules: identity block-ECA (IBE) and convolutional block-ECA (CBE). These modules incorporate a lightweight efficient channel attention (ECA) mechanism into traditional identity and convolutional residual modules. The ECA aims to enhance the network’s accuracy and sensitivity in feature extraction and analysis without altering the convolution operation process or feature map size. This integration significantly improves ERNet’s ability to recognize fine-grained features in hyperspectral corn seeds.
- We implemented effective cropping to optimize the utilization of ERNet in extracting finegrained features from hyperspectral corn seed images. This involved removing redundant backgrounds and enlarging the original image features. By employing this approach, we enhanced ERNet’s ability to extract detailed and precise features from the images.

The initial section will outline the characteristics of hyperspectral images and emphasize the significance of corn seed identification. The principles and advantages of efficient residual networks will be elaborated upon. The subsequent section will explain this article’s research objectives and methods, encompassing data collection and preprocessing, network model construction and training, and other relevant aspects. Finally, the study’s significance and anticipated results will be presented.

## 2 Related works

Extensive research has been conducted by scholars in seed classification, utilizing various methods categorized into traditional, machine learning, and deep learning approaches. The following provides an overview and summary of these research efforts.

Traditional methods have been attempted to be applied in seed recognition and hyperspectral image classification. Gan et al. (Gan et al., 2018) introduced a hyperspectral image classifier based on multi-feature kernel sparse representation. The features were transformed into a nonlinear low dimensional kernel space by employing kernel principal component analysis, enabling the handling of highly nonlinear distributions in hyperspectral image data. Experimental results demonstrated remarkable performance in hyperspectral image classification tasks. Hu et al. (Hu et al., 2020) showcased a promising technology that combined multispectral imaging and multivariate analysis. They utilized the LDA model to achieve 90% accuracy in alfalfa seed classification and SVM to achieve 91.67% accuracy in mycobacterium needle seed classification. Furthermore, Chen et al. (Chen et al., 2023) utilized the interior point hollowing algorithm to extract the outlines of sugarcane images on the MATLAB platform. They compared the effects of five classic edge detection operators on the same original sugarcane image and found the Canny operator to be the most suitable and effective. Li et al. (Li et al., 2023) proposed a method that combined terahertz time-domain spectroscopy (THz-TDS) imaging technology with the K-Means image segmentation method to detect the internal quality of pumpkin seeds accurately. Their approach achieved efficient results, with average detection errors of approximately 6.27% and 4.27% for single-frequency images at spatial resolutions of 0.4 mm and 0.2 mm, respectively. Ahmed et al. (Ahmed et al., 2020) conducted a study using X-ray imaging technology to investigate three watermelon varieties’ internal parameters (endosperm and air space). They evaluated traditional machine learning and deep learning methods and recognized X-ray imaging as promising.

These studies employed diverse hyperspectral image classification and feature extraction methods to accomplish seed identification. Nonetheless, traditional methods often focus on specific problems and datasets, which may limit their models’ and algorithms’ adaptability and generalization capabilities, warranting further improvement.

Machine learning methods provide solutions for algorithms and models to handle tasks such as seed recognition and hyperspectral image classification (Okwuashi and Ndehedehe, 2020; Chen et al., 2021a). Traditional crop seed classification and identification methods based on machine learning typically involve extracting features such as color, shape, texture, and others from images. These features are then used with classifiers like support vector machines (SVM) and artificial neural networks for classification purposes (Gao and Lim, 2019; Flores et al., 2021). For instance, Koklu et al. (Koklu and Ozkan, 2020). developed a computer vision system to differentiate seven dry bean varieties with similar characteristics. They employed image segmentation and feature extraction techniques, resulting in 16 features. By comparing the classification accuracy using 10-fold cross validation against four other methods, they found that the SVM classification model achieved the highest accuracy for bean variety classification. Su et al. (Su et al., 2020) utilized the KNCCRT integration framework and the random subspace (RS) concept to enhance diversity by randomly selecting features. They incorporated shape-adaptive (SA) neighborhood constraints within the RS integration framework to integrate spatial information. The method’s effectiveness was verified through experiments on three real hyperspectral datasets. In addition, Khatri et al. (Khatri et al., 2022) employed machine learning methods to classify wheat seeds based on seven physical characteristics. They observed 92% 94%, and 92% accuracy for KNN, decision tree, and naive bayes classifiers, respectively. An ensemble classifier based on hard voting achieved a maximum accuracy of 95% for decision-making. Zhang et al. (Zhang et al., 2020) utilized a random forest classifier along with multispectral data from Landsat 8 and Gaofen-1 (GF-1), field sample data, and panchromatic data from Gaofen-2 (GF-2). They calculated a time-series vegetation index from the data’s textural features and developed an RF classifier method for identifying corn seed fields. By inputting high-resolution remote sensing image features into this RF classifier, they successfully distinguished between two planting modes (seed and ordinary) and different types of corn varieties (selfing and hybrid), enabling the identification and mapping of extensive corn seed fields. Lastly, Ruslan et al. (Ruslan et al., 2022) proposed image processing and machine learning techniques were utilized to investigate the identification of weedy rice seeds. The researchers demonstrated that features extracted from RGB images, including color, morphology, and texture, exhibited higher sensitivity and accuracy compared to monochrome images.

In summary, researchers employ machine learning methods for crop seed identification as machine learning technology advances. These methods effectively identify crop seeds by extracting image features and utilizing various classifiers for classification. However, traditional machine learning methods often have high algorithm complexity and computational resource demands. This limitation hinders their real-time performance and scalability in practical applications.

Deep learning methods have made significant advancements and found widespread applications in agriculture. Researchers have utilized various methods to enhance the accuracy of image classification (Ding et al., 2020; Ding et al., 2023). These methods include the use of hybrid convolutional networks (Chen et al., 2020; Zhao et al., 2022a; Zhao et al., 2022b), innovative networks (Sun et al., 2023; Zhang et al., 2023b; Zhang et al., 2024b), improving image resolution (Paoletti et al., 2018; Liang et al., 2022), underwater image enhancement using different methods (Li et al., 2019; Li et al., 2021), multimodal deep learning models (Yao et al., 2023) and combining convolutional neural networks with hyperspectral images (Cao et al., 2020; Zheng et al., 2020; Xi et al., 2022; Yao et al., 2022). Deep learning methods address the limitations of traditional approaches by automatically learning feature representations from raw data, eliminating the need for manual feature design. They offer distinct advantages when dealing with complex and large-scale datasets.

With the ongoing development of deep learning, there is an increasing focus on applying deep learning techniques to seed classification tasks to enhance classification accuracy and robustness. For instance, Sellami et al. (Sellami et al., 2019) presented a novel approach for hyperspectral image (HSI) classification by integrating adaptive dimensionality reduction (ADR) and a semi-supervised three-dimensional convolutional neural network (3-DCNN). Their method effectively utilizes the deep spectral and spatial features extracted by convolutional encoder-decoders, substantially enhancing HSI classification accuracy. Zhang et al. (Zhang et al., 2021b) proposed a spectralspatial fractal residual convolutional neural network incorporating data balance enhancement. This method addresses the challenges posed by limited sample sizes and imbalanced categories, ultimately improving classification performance. Ahila et al. (Ahila Priyadharshini et al., 2019) developed a deep convolutional neural network based on an improved LeNet architecture to classify corn leaf diseases. By training their model on the PlantVillage dataset, they successfully classified it into four categories (three diseases and one healthy category) with an accuracy of 97.89%. Waheed et al. (Waheed et al., 2020) proposed an optimized dense convolutional network architecture for identifying and classifying corn leaf diseases. Their approach achieved an accuracy of 98.06% in accurately identifying and classifying these diseases. Furthermore, Javanmardi et al. (Javanmardi et al., 2021) proposed a novel method utilizing deep convolutional neural networks (CNN) as feature extractors. They employed multiple classifiers to classify the extracted features. Their findings demonstrated that the model trained on features extracted by CNN exhibited superior accuracy in classifying corn seed varieties, with the CNN-ANN classifier performing exceptionally well. Zhang et al. (Zhang et al., 2024a) proposed GACNet, a framework for wheat variety recognition. The framework includes semi-supervised generative adversarial networks for data augmentation and incorporates cross-conscious attention networks for variety recognition. GACNet achieves excellent classification performance through cross-learning of cascaded 3D and 2D convolutions. Li et al. (Guohou Li et al., 2024) used a hybrid convolutional neural network based on the attention mechanism to identify varieties of hyperspectral wheat, and applied a multivariate scattering correction method to attenuate spectral differences of the same variety due to differences in scattering levels. At the same time, principal component analysis was used to reduce the unwanted spectral bands of the three-dimensional data, and the classification accuracy of this method reached 97.92%.

Deep learning technology shows excellent potential in crop seed classification tasks. These studies provide new ideas and methods for the field of seed classification.

## 3 Methodology

**Figure 1:**
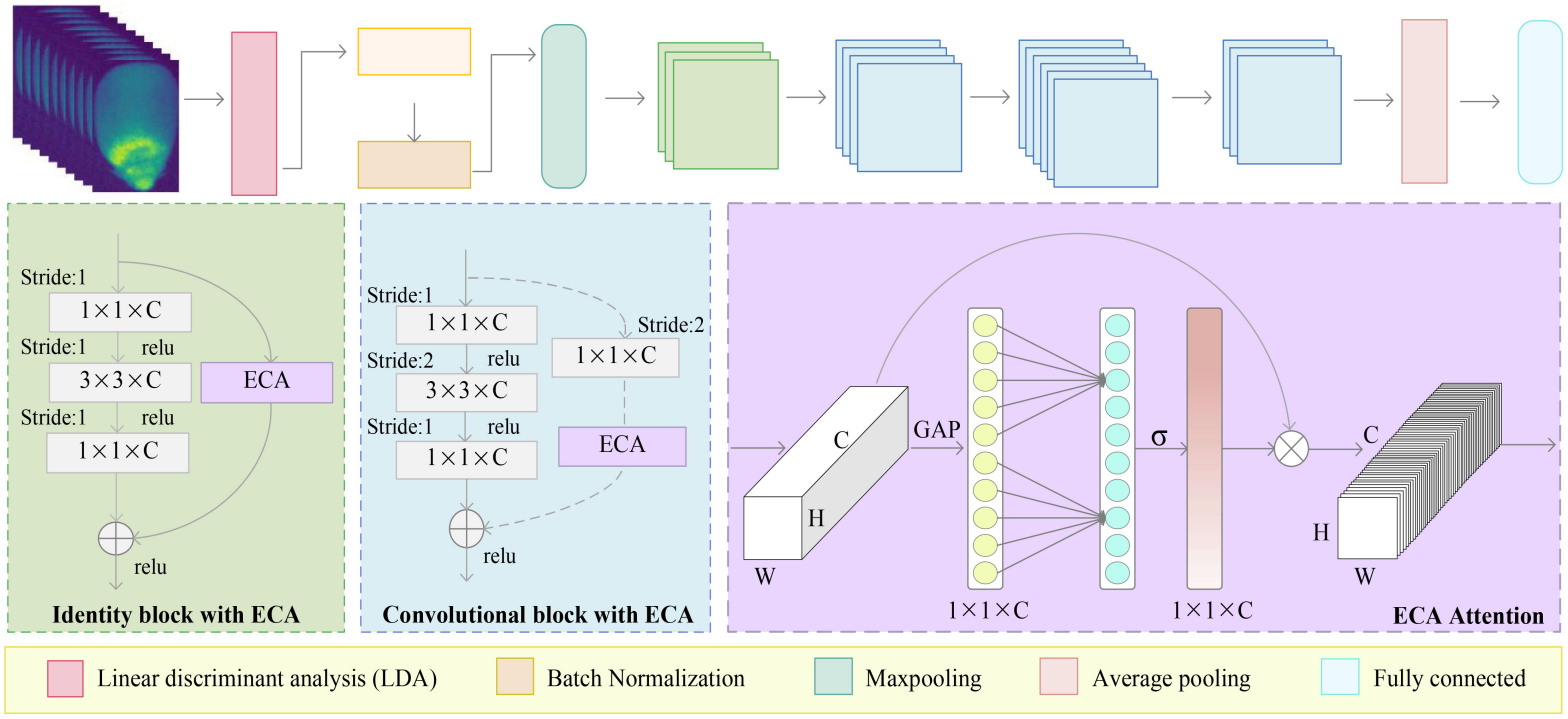
The flowchart of the ERNet method involves several steps. Initially, hyperspectral corn seed images undergo dimensionality reduction using LDA. Next, the images are subjected to convolutional operations for initial feature extraction. The texture features obtained are then refined using the efficient residual module to enhance their quality for the corn seed identification task.

The **Figure 1** illustrates the overall architecture of ERNet, designed for hyperspectral corn seed image classification. ERNet’s input stage receives standardized hyperspectral maize seed images. Subsequently, the hyperspectral images undergo dimensionality reduction using the linear discriminant analysis (LDA) module. The LDA module aims to extract discriminative features by maximizing inter-class mean differences and minimizing intra-class variance. ERNet incorporates an effective residual block called the E-R module, efficiently eliminating redundant data features and addressing uneven feature extraction issues. Finally, the extracted feature information is transformed into a fully connected feature vector, and the classification result is obtained in probability form using the Softmax function. This architecture empowers ERNet to process hyperspectral corn seed images effectively, extract discriminative features, and deliver accurate classification results. The process encompasses input processing, dimensionality reduction, feature extraction, and classification output, providing a professional and effective solution for hyperspectral image-based seed classification tasks.

### 3.1 Network framework

The **Figure 1** provides a detailed structure of ERNet, encompassing the LDA module, the efficient residual module, and the fully connected module. The LDA module framework is responsible for reducing dimensionality on hyperspectral images. The Efficient Residual module is employed to compact the network and extract precise features from hyperspectral corn seeds. Lastly, the fully connected module receives the extracted feature vector as input and employs a softmax classifier to compute class probabilities for the final classification result. Moreover, **Table 1** provides a comprehensive overview of each module within the ERNet model, highlighting their respective details.

**Table 1:** Details of each module of ERNet.

Layers (type)	Input Size	Output Size	Repeat	Parameter
Input	128 × 224 × 224	128 × 224 × 224	1	0
LDA	128 × 224 × 224	3 × 224 × 224	1	1280
Conv	3 × 224 × 224	64 × 112 × 112	1	9536
MaxPooling_1	64 × 112 × 112	64 × 56 × 56	1	0
IBE	64 × 56 × 56	256 × 56 × 56	3	232201
CBE_1	256 × 56 × 56	512 × 28 × 28	4	1086911
CBE_2	512 × 28 × 28	1024 × 14 × 14	6	7098422
CBE_3	1024 × 14 × 14	2048 × 7 × 7	3	14964763
Averagepooling	2048 × 7 × 7	2048 × 1 × 1	1	0
Fullyconnected	2048 × 1 × 1	2048 × 1 × 1	1	20490
Total trainable parameter:23413603				

### 3.2 Linear discriminant analysis module

Linear discriminant analysis (LDA) is a dimensionality reduction algorithm that leverages discriminant information within a given sample set. It constructs an intra-class scatter matrix to capture the variations among similar data samples and an inter-class scatter matrix to represent the differences between dissimilar data samples (Blei et al., 2003). By identifying an optimal projection direction, LDA is designed to minimize the intra-class scatter of similar data while maximizing the inter-class scatter of dissimilar data, thus achieving optimal separability among samples (Jia et al., 2021). Specifically, LDA transforms the sample data into a feature space using linear transformations, ensuring that samples of the same pattern type are closer to each other. In contrast, samples of different patterns are pushed farther apart. This mapping enables the extraction of discriminative features, which can serve as more informative inputs for subsequent classification tasks.

Let’s assume we have *S* training samples comprising *M* different pattern types, where the number of samples in each class is denoted as **S** *_i_* (*i* = 1,2,···, *M*). Class *M* is represented by $x_{i}=\{x_{i_{1}},x_{i},\cdots ,x_{i_{S_{i}}}\},x_{ij}(i=1,2,\cdots ,M;j=1,2,\cdots ,S_{i})$ is an n dimensional vector.

Consequently, we can compute the mean vector for each pattern type as Equation (1):


(1) $$v_{i}=\frac{1}{S_{i}}\sum_{j=1}^{S_{i}}x_{ij},$$

the total sample mean vector is Equation (2):


(2) $$v=\frac{1}{M}\sum_{i=1}^{M}v_{i}.$$

The intra-class scattering matrix **T** *_W_* and inter-class scattering matrix **T** *_B_* are respectively expressed as Equations (3) and (4):


(3) $$T_{W}=\sum_{i=1}^{M}\sum_{j=1}^{S_{i}}\left(x_{ij}-v_{i}\right){\left(x_{ij}-v_{i}\right)}^{T},$$

(4) $$T_{B}=\sum_{i=1}^{M}\left(v_{i}-v\right){\left(v_{i}-v\right)}^{T},$$

for any n-dimensional vector *a*, the function $f=\frac{a^{T}T_{B}a}{a^{T}T_{W}a}$ can be calculated. The function measures the linear separability between different pattern types by evaluating the ratio of the differences between dissimilar categories to the differences between similar types. A larger *f* value indicates a stronger linear separability, implying a higher discriminative power in distinguishing between different modes.

LDA effectively reduces data dimensionality while preserving the discriminative information between categories. By incorporating the LDA module, the classification performance in hyperspectral image seed classification can be enhanced, and redundant features can be minimized.

### 3.3 Efficient channel attention module

Studies have revealed that the channel attention mechanism effectively enhances the performance of neural networks (Shi et al., 2022). However, existing attention modules often exhibit complexity, which can lead to the problem of model overfitting. To tackle this problem, Wang et al (Wang et al., 2020) proposed a lightweight and versatile module called efficient channel attention (ECA). This study incorporates the ECA module into ERNet to assign channel weights to capture crucial features of hyperspectral corn seeds. Introducing the ECA module into ERNet enhances network performance and augments the ability to represent important features of hyperspectral corn seeds.

**Figure 2:**
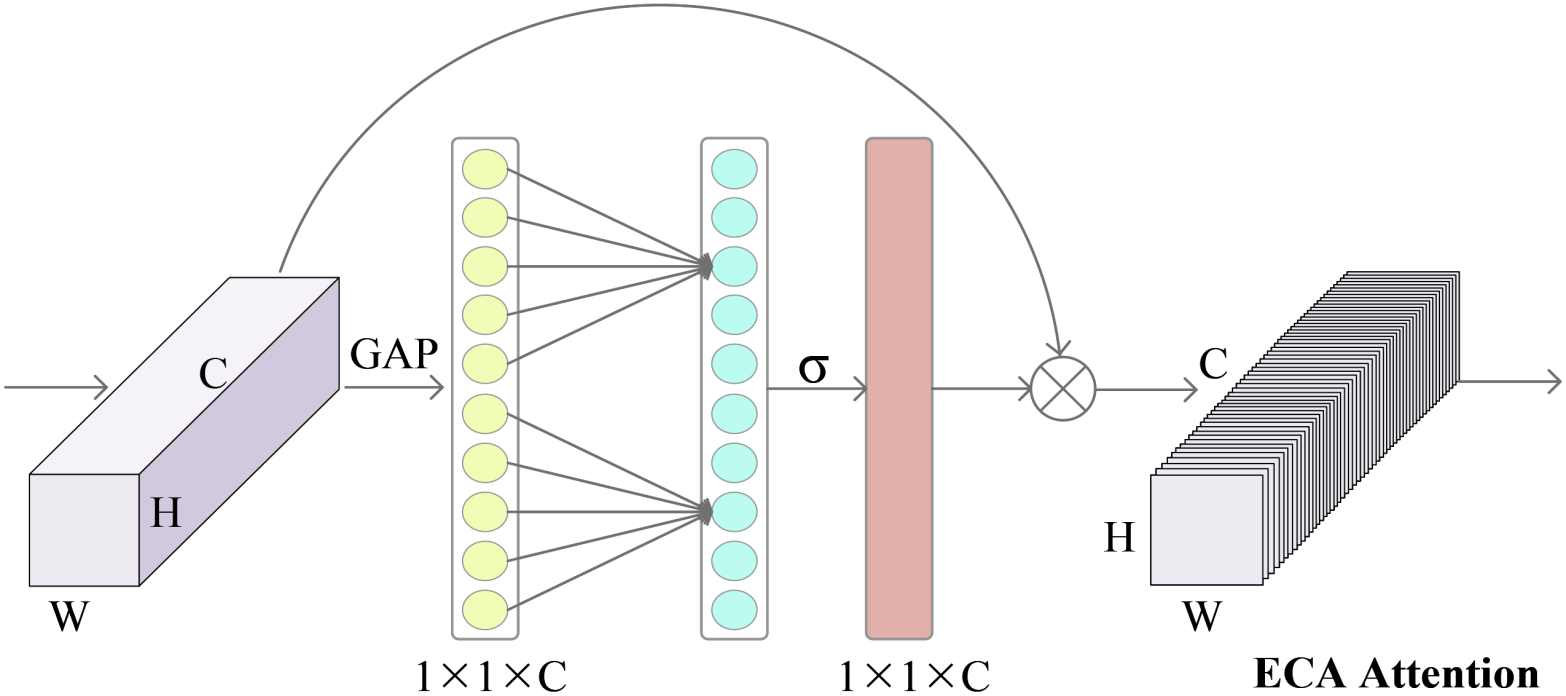
Feature refinement process of the efficient channel attention modules.

The **Figure 2** demonstrates the operational principle of the ECA channel attention mechanism. Global average pooling (GAP) is initially applied to the original input image to extract its features. This process involves averaging the features across each channel. Subsequently, the ECA module facilitates local cross-channel interactions through a rapid one-dimensional convolution operation, employing a kernel size denoted as k. Determining the convolution kernel’s size, k is adaptively achieved by leveraging a function that the number of input channels C. Following this, the sigmoid function is employed to assign weight proportions to each channel. These weights represent the significance of each channel in feature representation. Finally, the original input features are element-wise multiplied by the channel weights, resulting in a feature representation incorporating channel attention. Through these operations, the ECA module enables the network to prioritize crucial channels and extract discriminative image features.

In the ECA attention mechanism, the first step is to transform the dimension of the feature map obtained after the residual network and pooling at each scale. The feature map, initially in the shape of [H, W, C], is transformed into a vector of [1, 1, C]. Subsequently, the adaptive one-dimensional convolution kernel size, denoted as k, is calculated based on the channel number C of the feature map. Calculated as in Equation (5):


(5) $$\text{k}=\varphi \left(\text{C}\right)={\left|\frac{{\text{log}}_{2}C}{\gamma }+\frac{b}{\gamma }\right|}_{\text{odd}},$$

where *γ* takes value of 2, *b* takes value of 1, and odd takes odd number. The adaptive convolution kernel size, denoted as k, is calculated based on these values. The calculated k is then used for the one-dimensional convolution operation, which is applied to each channel of the feature map. The purpose of this operation is to capture the interactive information and reduce the degree of information loss between channels. Subsequently, the weights of each channel in the feature map are determined using the sigmoid function. The resulting consequences are then normalized, and the original input feature map is multiplied element-wise with the normalized weights to obtain the weighted feature map. This operation enables the network to prioritize essential channels, enhancing the features’ representation capabilities.

### 3.4 Efficient residual module

**Figure 3:**
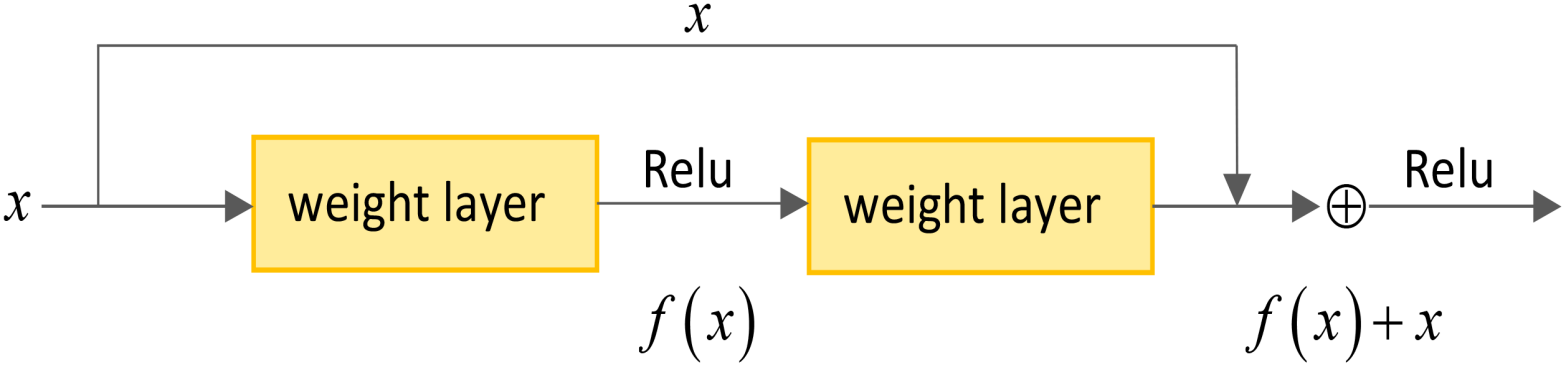
Basic unit legend of residual network.

The shortcut connections have been introduced into the residual network to facilitate optimization. A shortcut connection is a network structure that spans one or more layers and forms a residual learning unit by adding the input directly to the output. As depicted in the **Figure 3**, assuming the model input is denoted as *x*, and the original mapping as *R*(*x*), the core idea of residual learning is to design the network as *R*(*x*) = *f*(*x*) + *x*, where *f*(*x*) represents the residual mapping. A residual map *f*(*x*) + *x* is obtained by adding the residual map to the input. Although both mappings achieve the same expression effect, the residual map *f*(*x*) scale is relatively minor. Fitting *f*(*x*) is much simpler than doing the entire *R*(*x*) map. Replacing all the original mappings *R*(*x*) in the model with the residual mapping *f*(*x*) + *x*, reduces the difficulty of model fitting. The shortcut connections enable the network to learn the residual part more efficiently without excessively emphasizing the original mapping. This design more accessible training and optimization of the network, thereby enhancing the model’s performance and generalization ability.

In the context of the residual network, the output of each residual learning unit is denoted as *y_i_*, while the input is represented as *x_i_*. The mapping relationship within the residual learning unit can be expressed as Equation (6):


(6) $$y_{i}=f\left[x_{i}+H\left(x_{i},w_{i}\right)\right],$$

where the activation function *f* is applied within the residual learning unit. The rectified linear unit (ReLU) and the sigmoid function commonly use activation functions in neural networks. The term *H*(*x_i_,w_i_*) represents the residual, where *w_i_* represents the convolution kernel.

In the context of hyperspectral corn seed images, each image can be represented as a matrix *M* consisting of multiple column vectors. Matrix multiplication corresponds to a transformation, where a vector undergoes operations such as rotation or scaling to yield a new vector. When a matrix solely performs scaling or scales one or more vectors without introducing a rotational effect, these vectors are referred to as eigenvectors of the matrix, and the scaled value is known as the eigenvalue. Using methods such as gradient descent, the eigenvectors and eigenvalues of the matrix can be reversely fitted. These eigenvectors and eigenvalues are the characteristic information of hyperspectral corn seed images. We can classify and identify ideas by extracting and utilizing this feature information. The above transformation is formulated as Equation (7):


(7) M (X)=M (x)∗T (y),

where *M*(*X*) represents the matrix obtained after scaling transformation, *M*(*x*) denotes the original matrix, and *x* represents the column vector of the original matrix. *T*(*y*) represents a scaling matrix, where *y* signifies the scaling ratio applied to the column vector *x* within the matrix *M*.

**Figure 4:**
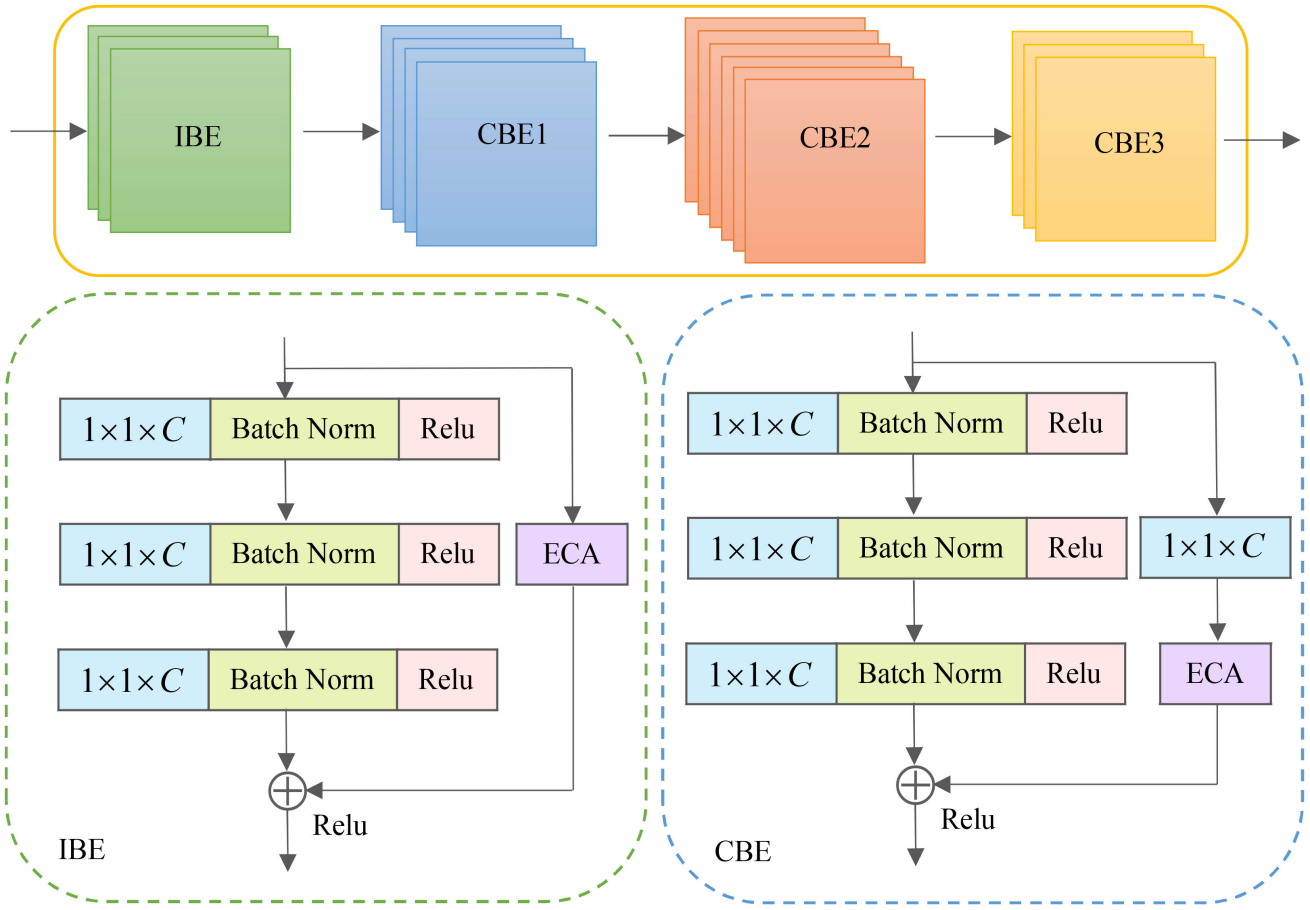
Feature refinement process of the efficient residual modules.

The **Figure 4** illustrates the efficient residual (ER) module, constructed by combining the IBE and CBE units. The number of stacks is determined through multiple tests, with the IBE module being stacked three times and the CBE module being repeated three times. Do 4, 6, and 3 stacks, respectively. Specifically, the basic unit comprises a sequence of cascaded operations, including convolution, batch normalization, activation function, convolution, batch normalization, activation function, convolution, batch normalization, and an ECA attention module. The pixel-by-pixel addition operation is employed within the basic unit. Additionally, short-circuit connections are incorporated within the basic unit to mitigate gradient vanishing issues and prevent network degradation.

The basic unit within the ER module enhances the network’s representation capabilities and improves the training process. It achieves this through cascaded convolution and batch normalization operations. The convolution operations aid in extracting essential image feature information and expand the network’s depth and receptive field, enhancing the model’s expressive ability. The batch normalization operation accelerates training and enhances the model’s robustness. Moreover, the activation functions introduce nonlinearity, allowing the network to capture complex relationships within the data.

To enhance the original model and improve its performance in image processing and computer vision tasks, we propose integrating the ECA module with the identity and convolutional blocks, respectively and refer to them as IBE and CBE structures. When the number of input and output channels of the essence or convolutional residual blocks is the same, we can directly incorporate an element-wise shortcut link by adding the input and output. This configuration forms the IBE structure. However, when the number of input and output channels differs in the identity or convolutional residual blocks, we introduce a convolution layer in the shortcut connection. This additional layer adjusts the dimension of the feature map to accommodate the disparity in input and output channel numbers, resulting in the formation of the CBE structure. We aim to enhance the original model and improve its performance in various image processing and computer vision tasks by employing these IBE and CBE structures.

### 3.5 Loss function

Cross entropy(CE) loss is a frequently used loss function in deep learning, especially in multiclassification problems. It draws upon concepts from information theory and measures the proximity between the actual output and the desired output. In information theory, the CE is utilized to estimate the average code length. In the context of deep learning, the CE loss function quantifies the dissimilarity between the model’s output’s probability distribution and the actual label’s probability distribution. A smaller CE value indicates a closer match between the two probability distributions. Given two probability distributions, *P_A_* (*x*) and *P_B_* (*x*), the CE between them can be expressed as Equation (8):


(8) $$\begin{array}{c} H\left(A,B\right)=\sum_{i=1}^{n}P_{A}\left(x_{i}\right)\log\;\frac{1}{P_{B}\left(x_{i}\right)}\\ =-\sum_{i=1}^{n}P_{A}\left(x_{i}\right)\log\;P_{B}\left(x_{i}\right), \end{array}$$

where *P_A_* (*x*) represents true label distribution in the given expression, while *P_B_* (*x*) represents the predicted distribution. As a measure, the CE quantifies the disparity between the expected value and the actual label value. More precisely, the CE loss function gauges the uncertainty of the predicted distribution about the actual distribution. To measure the distance and dissimilarity between two probability distributions, kullback-leibler divergence (KL-divergence) is employed. The KL-divergence is represented as Equation (9):


(9) $$\begin{array}{c} D_{KL}\left(A∥B\right)=\sum_{i=1}^{n}P_{A}\left(x_{i}\right)\log\;\frac{P_{A}\left(x_{i}\right)}{P_{B}\left(x_{i}\right)}\\ =\sum_{i=1}^{n}P_{A}\left(x_{i}\right)\log\;P_{A}\left(x_{i}\right)-\sum_{i=1}^{n}P_{A}\left(x_{i}\right)\log\;P_{B}\left(x_{i}\right)\\ =-H\left(A\right)+H\left(A,B\right), \end{array}$$

where $D_{KL}\left(A∥B\right)$ achieves its minimum value only when *P_A_* (*x*)=*P_B_* (*x*), indicating that the closer the predicted result is to the actual result better. The CE loss function is a specific instance of KL-divergence and finds extensive application in deep learning’s multi-classification problems. We aim to minimize the CE loss function to make the predicted *P_B_* (*x*) as similar as possible to the actual label distribution *P_A_* (*x*). This alignment ensures that the model’s predictions are consistent with the results.

The multi-class CE loss function serves as the evaluation criterion for the model. The network aims to minimize the CE by updating the weights of its nodes. To achieve this, the model employs the stochastic gradient descent algorithm. This algorithm optimizes the loss function to determine the optimal parameters and minimize the loss. The optimization process of the stochastic gradient descent algorithm can be defined as Equation (10):


(10) $$\theta_{i}=\theta_{j}-\alpha *\frac{\partial J\left(\theta \right)}{\partial \theta },$$

where *θ_i_* represents the weight of the current network node, *θ_j_* denotes the weight from the previous iteration of the network, and *α* represents the learning rate of the model. During each iteration, the model optimizes *θ* through gradient descent, aiming to minimize the CE. The goal is to reach the lowest possible the CE, enabling the entire model to converge toward the global optimal solution.

## 4 Experiments

This chapter begins by introducing the dataset utilized in the study. It then proceeds to describe the training process of ERNet, followed by conducting comparative and ablation experiments to demonstrate the significance of ERNet in hyperspectral corn seed classification. The results obtained from these experiments provide valuable insights and reference points for evaluating the effectiveness of ERNet in the classification task.

### 4.1 Dataset used

**Figure 5:**
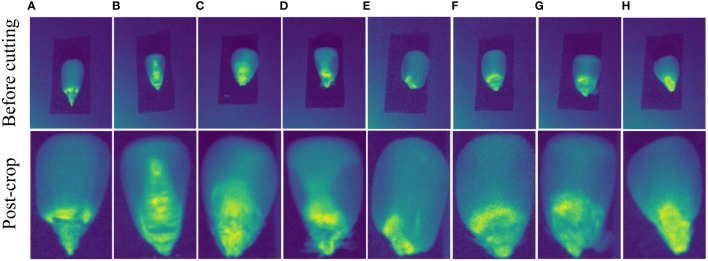
The provided images showcase different types of seed images. The pictures labeled “before cutting” are sourced from SSTNet, while those labeled “after cropping” have been manually and accurately cropped. The above eight sets of images correspond to the following corn varieties: **(A)** baiyu 607, **(B)** baiyu 808, **(C)** baiyu 818, **(D)** baiyu 833, **(E)** baiyu 8317, **(F)** baiyu 9284, **(G)** baiyu 897, and **(H)** fengda 601.

We method run on a Windows 10 PC with AMD Ryzen 5 3600X Central Processing Unit (CPU) at 3.80 GHz, The dataset (CSHID) utilized in this article is sourced from SSTNet (Zhang et al., 2022), encompassing ten different corn varieties cultivated in Henan Province: Baiyu 607, Baiyu 808, Baiyu 818, Baiyu 833, Baiyu 879, Baiyu 897, Baiyu 918, Baiyu 8317, Baiyu 9284, and Fengda 601. The data was collected using Surface Optics’ SOC 710 Portable Visible/Near Infrared Imaging Spectrometer. Each corn variety consists of 120 samples, with each piece containing 128 spectral bands. The original spectra were precisely cropped to ensure accuracy, resulting in 129,230 sample images employed in this study. The **Figure 5** showcases a comparison of selected images before and after cropping.

### 4.2 Experimental settings

The experimental setup for this article consisted of a computer equipped with an AMD Ryzen 7 5800H with Radeon Graphics CPU, operating at 3.20GHz and 16GB of RAM. Additionally, it included an NVIDIA GeForce GTX 1650 graphics card with 4GB of video memory. The software environment for the experiments involved Python 3.7.13 and torch-gpu-1.10.1, running on the 64-bit Windows 11 operating system.

The fully connected layer incorporates dropout technology to prevent overfitting during model training. Additionally, the model’s parameters are optimized using the Adam optimizer. Classification results determine a learning rate of 0.01 as the optimal choice. Furthermore, an exponential decay learning rate enhances model stability during later training. This approach gradually reduces the learning rate over time. The training process follows a batch training method with a batch size of 32. Batch training involves dividing the training dataset into several batches, each containing a specific number of samples. The model performs forward propagation and back propagation calculations on each set to update the parameters. After 150 iterations, the loss rate stabilizes, indicating that the model has converged and achieved relatively stable performance.

### 4.3 Identification evaluation

When it comes to deep learning, more data is often required for practical training than traditional machine learning approaches. This paper randomly divides the dataset into a training set and a test set following a “training set: test set = 4:1” principle. Four machine learning and six deep learning models are selected as reference models to conduct comparative experiments. The machine learning models consist of fuzzy k-nearest neighbor (FKNN) (Kumbure et al., 2020), random forest algorithm (RFA) (Chen et al., 2021b), stochastic gradient descent (SGD) (Lei and Tang, 2021), and spatial-spectral feature extraction method (FSVM) (Jin et al., 2022). The deep learning models include hybrid spectral net (HybridSN) (Roy et al., 2019), centernet (Jin et al., 2021), spatial source phase net (SSPNet) (Lin et al., 2022), spatial, spectral, and texture aware attention network (SSTNet) (Zhang et al., 2022), convolutional neural network with a bidirectional gated recurrent unit (CNN-BiGRU) (Lu et al., 2023), and Convolutional Neural networks with long short-term memory (CNN-LSTM) (Wang and Song, 2023).

Model performance is assessed using four metrics: F1 score, recall, precision, and accuracy. Accuracy measures the correct classification rate of both positive and negative samples. Precision is the ratio of true positives to all positive classifications. Recall measures the percentage of correctly classified positive models out of all positive examples. The F1 score is a comprehensive evaluation index that combines precision and recall. Higher values of these metrics indicate better classification performance. By comparing the metric results across different models, their effectiveness in classification tasks can be evaluated. We used the same test sets and training parameters in comparison tests to assess ERNet against several different approaches. The outcomes, as reported in **Table 2**.

**Table 2:** Identification results of different deep learning methods tested on the CSHID dataset.

Method	F1-score	Recall	Precision	Accuracy
FKNN (Kumbure et al., 2020)	0.9610	0.9583	0.9637	0.9625
RFA (Chen et al., 2021b)	0.9426	0.9411	0.9435	0.9479
SGD (Lei and Tang, 2021)	0.9585	0.9663	0.9703	0.9705
FSVM (Jin et al., 2022)	0.9467	0.9417	0.9519	0.9458
HybridSN (Roy et al., 2019)	0.9621	0.9667	0.9673	0.9708
CenterNet (Jin et al., 2021)	0.9253	0.9255	0.9260	0.9258
SSPNet (Lin et al., 2022)	0.9695	0.9695	0.9696	0.9693
SSTNet (Zhang et al., 2022)	0.9795	0.9791	0.9800	0.9792
CNN-BiGRU (Lu et al., 2023)	0.9396	0.9385	0.9384	0.9393
CNN-LSTM (Wang and Song, 2023)	0.9497	0.9501	0.9534	0.9509
ERNet	0.9833	0.9830	0.9846	0.9836

(Optimal: red; Suboptimal: blue).

RFA (Chen et al., 2021b) employs the random forest algorithm to compute variable importance and weights for security risk indicators, demonstrating high accuracy on large-scale datasets. FSVM (Jin et al., 2022) utilizes principal component analysis to extract features from spatial-spectral data and trains and optimizes the model using support vector machines, resulting in good classification performance on small sample datasets. FKNN (Kumbure et al., 2020) utilizes local mean vectors and Bonferroni means, showcasing strong performance despite significantly imbalanced data class distributions. SGD (Lei and Tang, 2021) introduces high-probability bounds on computational and statistical errors, enabling the development of a new learning rate for non-convex learning with SGD by adjusting the number of passes to balance these errors. SSPNet (Lin et al., 2022) utilizes spatial source phase (SSP) maps derived from complex-valued fMRI data as input for CNN and achieves noteworthy results in image recognition. HybridSN (Roy et al., 2019) and SSTNet (Zhang et al., 2022) are hybrid CNN models that jointly leverage 3D-CNN to represent spatial-spectral features from spectral bands. SSTNet additionally incorporates a spatial channel attention mechanism. Both methods deliver satisfactory performance in hyperspectral image classification. CenterNet (Jin et al., 2021) combines deep learning and image processing techniques, utilizing genetic algorithms to determine indicators and evaluate results, resulting in commendable classification performance. CNN-BiGRU (Lu et al., 2023) combines a convolutional neural network with a bidirectional gated recurrent unit, introducing residual mechanisms and an improved convolutional attention module, demonstrating promising outcomes in rice disease identification. CNN-LSTM (Wang and Song, 2023) combines a convolutional neural network (CNN) with a long short-term memory (LSTM) network and achieves accurate identification of corn varieties in conjunction with hyperspectral imaging technology. Nevertheless, the classification results obtained by these traditional and deep learning methods still lower than ERNet.

**Table 2** makes it clear that when compared to other techniques, the machine learning models RFA (Chen et al., 2021b) and FSVM (Jin et al., 2022) perform worse in classification. RFA (Chen et al., 2021b) and FSVM (Jin et al., 2022) perform somewhat worse in classification than FKNN (Kumbure et al., 2020) and SGD (Lei and Tang, 2021). Although deep learning techniques like CNN-BiGRU (Lu et al., 2023), CNN-LSIM (Wang and Song, 2023), and CenterNet (Jin et al., 2021) have considerable benefits, their classification performance isn’t perfect. HybridSN (Roy et al., 2019), SSPNet (Lin et al., 2022), and SSTNet (Zhang et al., 2022) do not outperform our ERNet on a variety of indicators, even though they take into account spectral spatial information and perform well in classification. In conclusion, our ERNet performs exceptionally well in classification due to its superiority in picture feature extraction. Regarding overall performance, the ERNet network demonstrates notable advantages across all evaluation metrics. The accuracy achieved by the ERNet network reaches an impressive 98.36%. The accuracy improvement ranges from 1.31% to 3.78% compared to the machine learning models. Similarly, the other deep learning models show accuracy gains ranging from 0.44% to 3.27%. These results highlight the significant enhancement in corn hyperspectral image classification accomplished by the ERNet network.

**Figure 6:**
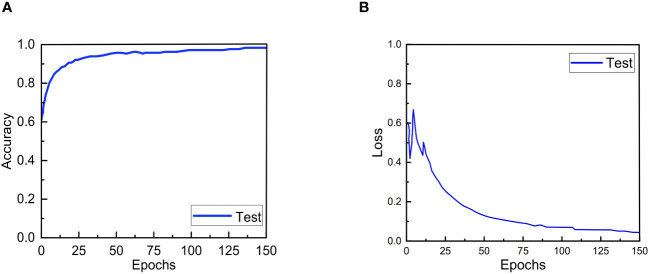
Accuracy and loss convergence over the number of epochs on the test set. **(A)** Accuracy convergence over the number of epochs. **(B)** Loss convergence over the number of epochs.

The accuracy and loss convergence curves of ERNet during testing are shown in The **Figures 6A, B**. It is clear that ERNet exhibits faster convergence before 20 epochs, and by the 130th epoch, it has achieved good convergence and high accuracy. After more than 130 epochs, ERNet stabilizes.

Comparing the training time of each model on the CSHID dataset, it can be seen from **Table 3** that ERNet outperforms traditional machine learning with the latest network models for training hyperspectral images in terms of training time, which shows that the ERNet model achieves an excellent balance between efficiency of use and improvement in accuracy, specifically through the advantages of combining residual networks with lightweight attention mechanisms to achieve network performance improvement.

**Table 3:** Training and testing times on the CSHID dataset, training times are based on one epoch.

Method	Training time(s)	Testing time(min)
FKNN (Kumbure et al., 2020)	25.7	20.3
RFA (Chen et al., 2021b)	35.4	28.4
SGD (Lei and Tang, 2021)	26.0	28.3
FSVM (Jin et al., 2022)	34.6	27.8
HybridSN (Roy et al., 2019)	37.1	29.7
CenterNet (Jin et al., 2021)	46.6	31.8
SSPNet (Lin et al., 2022)	37.6	32.4
SSTNet (Zhang et al., 2022)	26.4	29.7
CNN-BiGRU (Lu et al., 2023)	26.2	23.4
CNN-LSTM (Wang and Song, 2023)	35.9	26.8
ERNet	**20.3**	**19.6**

The test time is the result of categorizing the entire dataset. (Optimal results are bolded.)

### 4.4 Ablation study

Ablation experiments were performed to evaluate the effectiveness of each module in ERNet for hyperspectral maize seed detection. The following ablation operations were performed on ERNet individually: 1) our ERNet without efficient channel attention module (-w/o ECA); 2) our ERNet without convolutional block-ECA (-w/o CBE); 3) our ERNet without identity block-ECA (-w/o IBE). The ablation experiments enabled a thorough evaluation of the effect of each module on the performance of ERNet in recognizing hyperspectral corn seeds.

**Table 4** presents the f1-score, recall, and accuracy results for each ablation experimental model and the corresponding accuracy score for the full ERNet model. By comparing the practical outcomes, it is evident that the complete ERNet model achieved the highest scores across all metrics compared to the ablation models.

**Table 4:** Results of different modules for the implementation of ablation studies on test samples may exhibit discriminatory tendencies.

Method	F1-score	Recall	Precision	Accuracy
-w/o ECA	0.9570	0.9578	0.9572	0.9571
-w/o CBE	0.9354	0.9341	0.9343	0.9351
-w/o IBE	0.6432	0.6387	0.6359	0.6328
ERNet (full model)	0.9833	0.9830	0.9846	0.9836

(Optimal: red; Suboptimal: blue).

## 5 Discussion

The research presented in this article holds significant importance for corn seed identification within the agricultural domain. By leveraging an efficient residual network to process high-dimensional hyperspectral image data, the accuracy and efficiency of corn seed identification can be substantially enhanced. This, in turn, enables precise planting management and facilitates advancements in crop varieties for agricultural production. The intended outcome of this research is to demonstrate experimentally that features within hyperspectral images can be effectively extracted using an efficient residual network, leading to accurate classification and identification of corn seeds. Furthermore, this article’s research methods and findings can serve as a valuable reference for studying hyperspectral image recognition and classification in other crop-related research endeavors.

Future challenges include realizing complete seed screening in the recognition process and using hyperspectral technology for maturity discrimination to achieve a true sense of superior breed recognition. These challenges are worthwhile to pursue in order to develop more functional deep learning models for seed recognition in a variety of scenarios.

## Data availability statement

The original contributions presented in the study are included in the article/supplementary material. Further inquiries can be directed to the corresponding author.

## Author contributions

XYL: Writing – review & editing. MJZ: Writing – original draft, Writing – review & editing. LYZ: Writing – review & editing. LZ: Formal analysis, Writing – review & editing. XWX: Writing – review & editing. WYZ: Data curation, Writing – review & editing. WDZ: Methodology, Writing – review & editing.
